# Initial outcomes with uniportal video-assisted lung resection

**DOI:** 10.1093/icvts/ivaf111

**Published:** 2025-05-08

**Authors:** James Shahoud, Benny Weksler, Brent Williams, Lawrence Crist, Hiran Fernando

**Affiliations:** Department of Surgery, Allegheny General Hospital, Pittsburgh, PA, USA; Department of Thoracic and Cardiovascular Surgery, Division of Thoracic and Esophageal Surgery, Allegheny General Hospital, Pittsburgh, PA, USA; Department of Thoracic and Cardiovascular Surgery, Division of Thoracic and Esophageal Surgery, Allegheny General Hospital, Pittsburgh, PA, USA; Department of Thoracic and Cardiovascular Surgery, Division of Thoracic and Esophageal Surgery, Allegheny General Hospital, Pittsburgh, PA, USA; Department of Thoracic and Cardiovascular Surgery, Division of Thoracic and Esophageal Surgery, Allegheny General Hospital, Pittsburgh, PA, USA

**Keywords:** uniportal video-assisted thoracic surgery, lung cancer, lobectomy, segmentectomy, surgical outcomes

## Abstract

**OBJECTIVES:**

Uniportal video-assisted lung resection is increasingly reported, but adoption in North America has been low. This study examines the early experience with the uniportal technique by a surgeon experienced in multiportal thoracoscopic surgery.

**METHODS:**

Operation was performed using a 4-cm incision crossing the anterior axillary line. Primary objectives were to evaluate short-term outcomes, and secondary objectives included evaluation for learning curve and oncological outcomes for patients with non-small cell lung cancer.

**RESULTS:**

Over a 45-month period, 212 patients underwent uniportal lung resection. Procedures included 128 lobectomies (60.4%), 41 segmentectomies (19.3%), 40 wedge resections (18.9%) and 3 extended resections (1.4%). Conversion was required in 24 patients (12.8%); 17 to multiportal surgery and 7 to thoracotomy. Major adverse events occurred in 13 patients (6.1%) and included 3 deaths (1.4%). Median hospital stay was 3 days, and median chest tube duration was 2 days. When comparing the early and late experience, there was no difference in hospital stay, adverse events, conversion and readmissions. The mean number of lymph nodes and nodal stations dissected were 10.08 and 4.79, respectively. The number of nodal stations dissected improved with experience (*P* < 0.001).

**CONCLUSIONS:**

Uniportal video-assisted lung resection is safe with good perioperative outcomes. Lymph node dissection improved with experience; otherwise, no significant learning curve was demonstrated when transitioning from a multiport approach.

## Graphical Abstract

**Figure:**
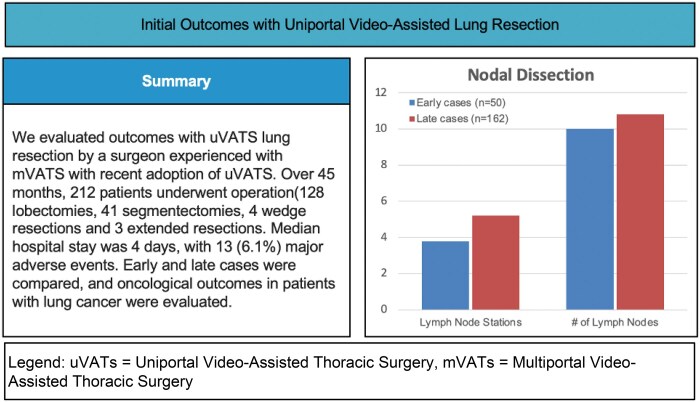

## INTRODUCTION

For over two decades, video-assisted thoracic surgery (VATS) has been increasingly adopted by thoracic surgeons for the surgical treatment of lung cancer. The most common approach has been a multiport VATS (mVATS) approach [1]. More recently, there has been interest in other minimally invasive approaches such as robotic, uniportal and sub-xiphoid VATS [2–4]. Uniportal VATS (uVATS) was first reported in 2004 by Rocco *et al.* for wedge resection of the lung [5]. In 2011, Gonzalez-Rivas *et al.* reported on lobectomy using a uVATS approach [3]. Since then, there has been increasing adoption of uVATS, particularly among centres in Asia and Europe. For surgeons who are experienced with a multiport minimally invasive approach, there has been a reluctance to undertake the learning curve, to adopt uVATS. In part, this may relate to a lack of comparative studies available to compel a change to their operative approach. Suggested advantages of uniportal over multiportal VATS for anatomical resections include less intraoperative blood loss, reduced postoperative pain and shortened hospitalization [6].

In this study, we report our initial experience with uVATS from a North American centre. Operations were performed by a surgeon with over 15 years’ experience performing mVATS lobectomy, and with recent adoption of uVATS. In preparation for transitioning to uVATS, the surgeon undertook a sabbatical at Shanghai Pulmonary Hospital, a high-volume centre where several uVATS pulmonary resections are routinely performed every day [7]. This experience was very helpful in understanding instrument positioning through the uniport incision, and the differences in camera view compared to an mVATS approach. In mVATS, the usual view is with the apex of the chest at the top (12-o’clock position) of the screen, and the diaphragm at the bottom (6 o’clock position). Instruments are triangulated to the target anatomy through the various port incisions. With uVATS, the usual camera view is a side-to-side view with the spine at the top of the screen (12 o’clock position) and the anterior aspect of the chest at the bottom of the screen (6 o’clock position). Although instruments are not automatically triangulated in the same way as mVATS because of the nature of port placement, freedom of movement is achieved by having the surgeon’s instruments enter the uniport incision from opposite angles (e.g. right hand instrument from right to left, and left hand instrument from left to right). The initial cases in the study were performed by the attending surgeon, but later on were increasingly performed by residents.

## MATERIALS AND METHODS

### Study design

This was a retrospective study that was approved by our institutional review board. Operations were performed over a 45-month period from July 2020 to March 2024. All patients who underwent therapeutic wedge resection, segmentectomy or lobectomy by a uVATS approach during this time-frame were included. Patients requiring a diagnostic wedge resection for suspected interstitial lung disease, apical wedge resection for pneumothorax or lung decortication were excluded. The primary objectives were to evaluate short-term outcomes (adverse events, length of stay, chest-tube duration). Secondary objectives included evaluation for learning curve by comparing patients operated on in the early and later experience, and oncological outcomes for patients with non-small cell lung cancer (NSCLC).

### Operative technique

A 4 cm access incision is typically made crossing the anterior axillary line in the fourth interspace for upper and middle lobe resections, and the fifth interspace for lower lobe resection. The surgeon stands anterior and the assistant, posterior to the patient. A wound protector is then placed in the incision. A 30-degree 5 mm camera is placed in the posterior aspect of the incision by the assistant, and the main working instrument for the surgeon (e.g. sealer-dividing instrument, dissecting instruments, stapler) is placed in the anterior aspect of the incision by the surgeon. We do not use an epidural. A multi-level intercostal block using bupivacaine is always performed. We typically use 20 cc of 0.25% bupivacaine divided between five intercostal spaces. A single 24f Blake drain is used for a chest tube. Criteria for chest-tube removal are the absence of an air leak, and drainage of 450 ml or less over a 24-hour period.

### Outcome measures and statistical analysis

Adverse events were recorded and included major adverse events as defined in the Society of Thoracic Surgeons (STS) General Thoracic Database. Prolonged air leak was defined as chest tube duration greater than 5 days or discharge with a chest tube. Statistical analysis was performed using SPSS Version 29, employing descriptive statistics to summarize patient demographics, clinical characteristics and surgical outcomes. To assess learning curve effect, regression analysis was undertaken. To determine associations between case number (e.g. first case, second case, etc.) and case complexity and outcomes, regression models were developed with case number as the sole independent variable with either continuous (linear regression) or binary (logistic regression) dependent variables. These models were tested for linear trends in the respective outcomes as the case number increases. Kaplan–Meier plots and log-rank tests evaluated overall survival (OS) and recurrence rates among NSCLC patients.

## RESULTS

The study included 212 patients. There were 89 (42%) men and 129 (58%) women. Mean age was 68 (37–90) years, and mean body mass index (BMI) was 28 (15–52). Mean forced expiratory volume in 1 second (FEV1%) was 83.7%, and mean diffusing capacity of the lungs for carbon monoxide (DLCO) was 78.5%. The procedures performed included 128 lobectomies (60.4%), 41 segmentectomies (19.3%), 40 wedge resections (18.9%) and 3 extended resections (1.4%). The extended resections included two patients who required bilobectomy and one patient who required lobectomy with partial diaphragm resection. Seventeen patients (8%) required conversion to mVATS, and seven patients (3%) required conversion to open surgery. The decisions to convert to an open procedure were made due to bleeding from the pulmonary artery, difficulty tolerating single-lung ventilation or inadequate visualization. Adverse events of any severity occurred in 70 patients (33%) and major adverse events occurred in 13 patients (6.1%). This included pneumonia (one case), pulmonary embolism (two cases), myocardial infarction (one case), unexpected return to the operating room (six cases) and mortality (three cases). Thirty-day mortality occurred in three patients (1.4%). The median length of stay was 3 days, and the median chest tube duration was 2 days. Prolonged air leak occurred in 33 patients (15.6%) and 14 (6.6%) were discharged home with chest tubes.

**Figure 1:**
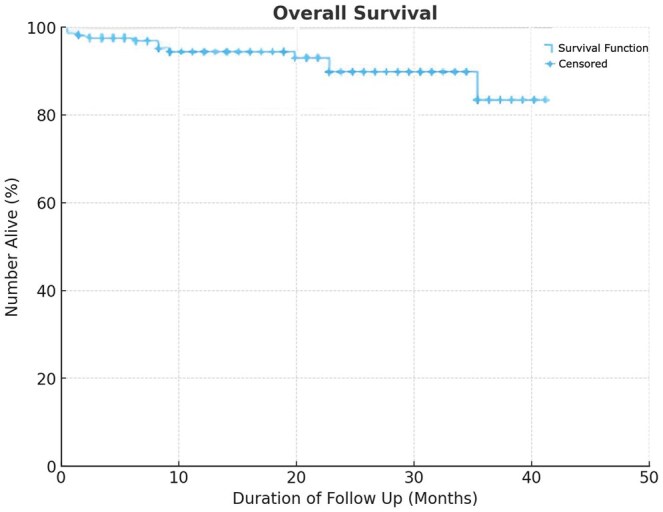
Overall survival curve of patients post-procedure using Kaplan–Meier method, with survival duration measured in months. Vertical tick marks indicate censored data points, representing patients who were alive at the end of their follow-up period or lost to follow-up

**Figure 2:**
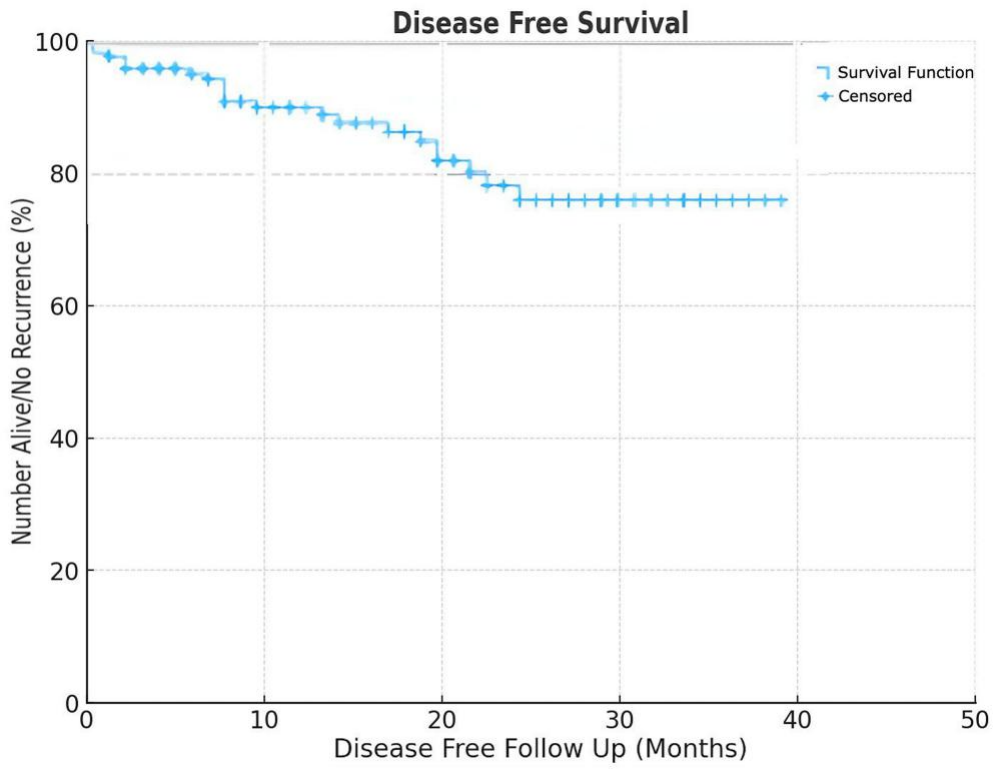
Disease-free survival of patients post-procedure using Kaplan–Meier method

There were 50 patients (23.6%) with either benign lung pathology or metastatic disease from a different primary tumour. We separately analysed the 162 patients (76.4%) in our study specifically with primary lung cancer. Of these, ten patients (6.2%) received neoadjuvant chemotherapy/immunotherapy. Procedures performed included lobectomy (*n* = 117; 72.3%), segmentectomy (*n* = 31; 19.1%), wedge resection (n= 13; 8%), and one bilobectomy (0.6%). Pathological stages were I (*n* = 126), II (*n* = 24) III (*n* = 11) and IV (*n* = 1). The mean number of lymph nodes and stations dissected were 10.5 and 4.9, respectively. The mean follow-up period was 16.46 months, and the overall recurrence rate was 5.6%. OS and disease-free survival (DFS) are displayed in Figs 1 and 2, respectively.

**Figure 3:**
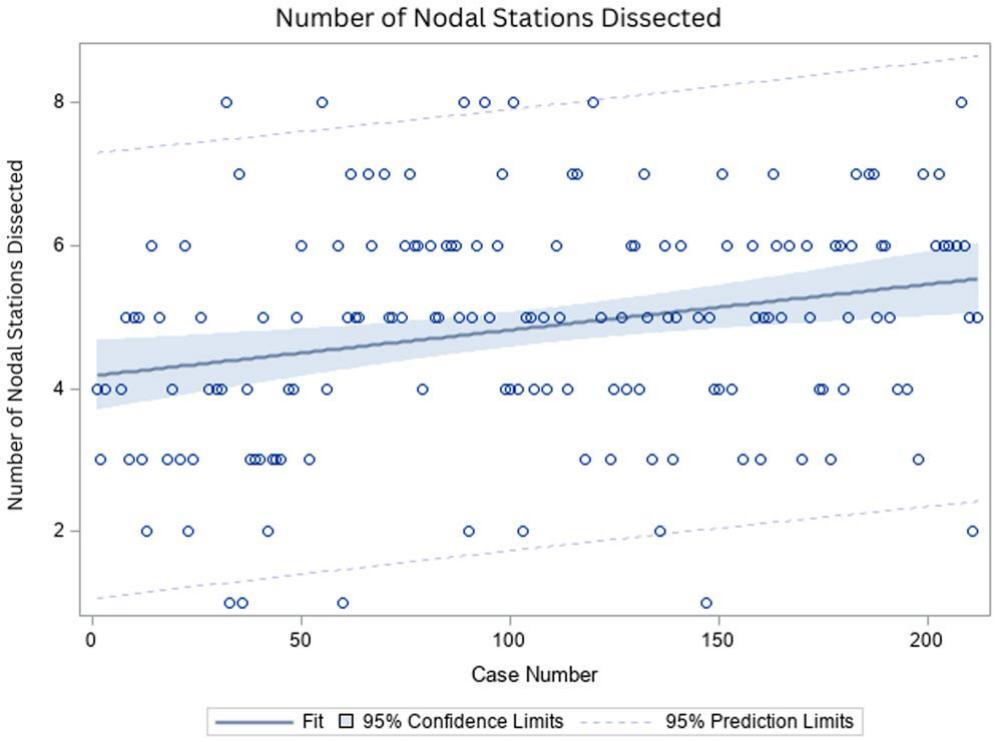
Regression analysis demonstrating an increasing number of nodal stations dissected as procedure count increases

Comparisons between patients operated on in the first, second and third part of our experience are outlined in Table 1. Patients had similar baseline demographics. We saw a significant improvement in the number of nodal stations dissected with experience (Fig. 3). Additionally, there was reduction in the number of patients discharged with a chest tube. There was however an increase in the length of stay by one day.

**Table 1:** Analysis of early case series versus later case series; comparing demographics, operative times and surgical outcomes in patients undergoing uVATS

	First third (*n* = 71)	Second third (*n* = 70)	Last third (*n* = 71)	*P* value
Age	69.5 (9.8)	68.1 (9.0)	68.4 (8.9)	0.45
BMI	27.4 (5.9)	28.6 (6.2)	28.2 (6.1)	0.54
FEV%	82.2 (18.5)	84.7 (22.4)	84.5 (18.9)	0.53
DLCO%	76.5 (25.7)	81.5 (22.9)	77.5 (21.5)	0.79
Segmentectomy performed	8 (11%)	15 (21%)	18 (25%)	0.06
Procedure time (min)	177.6 (72.1)	177.6 (67.2)	174.1 (67.0)	0.82
Number of lymph nodes dissected	10.5 (7.0)	10.1 (5.2)	11.0 (6.6)	0.45
Number of lymph node stations dissected	4.2 (1.7)	5.2 (1.5)	5.1 (1.5)	0.002
Conversion to mVATS/open	8 (11%)	9 (13%)	7 (10%)	0.85
Conversion to open	2 (3%)	4 (6%)	1 (1%)	0.95
Any complication	28 (39%)	22 (31%)	20 (28%)	0.53
Major complication	4 (6%)	3 (4%)	6 (8%)	0.35
30-day mortality	0 (0%)	1 (1%)	2 (3%)	0.23
Prolonged air leak	11 (15%)	14 (20%)	10 (14%)	0.86
Mean chest tube duration (days)	3.9 (5.7)	3.4 (3.7)	4.0 (5.7)	0.18
Mean length of stay (days)	3.3 (2.3)	3.8 (3.6)	4.3 (3.0)	0.03
Discharged with chest tube	7 (10%)	6 (9%)	1 (1%)	0.04
30-day readmission	5 (7%)	5 (7%)	9 (13%)	0.26

## DISCUSSION

Since 1992, VATS has become increasingly utilized for the treatment of NSCLC [8]. Randomized studies as well as meta-analyses have demonstrated benefits of mVATS compared to thoracotomy with less pain, improved quality of life and lower complication rates [9–11]. uVATS has emerged as a popular minimally invasive approach in many countries, but with relatively little uptake among North American Surgeons. Gilbert *et al.* published results from a survey conducted on 600 thoracic surgeons in North America. They found that 70% of surgeons had never performed uVATs and that they would require scientific evidence of superiority or superior ergonomics to consider this technique [12]. Theoretically, operating through a single anteriorly placed incision should result in less pain compared to multiple incisions through different interspaces. Additionally avoiding a posterior based incisions may be preferable as the intercostal spaces are narrower, and trauma to the intercostal nerve more likely. If pain is less, this may allow improved ability to cough, clear secretions and ambulate potentially leading to a lower incidence of complications. Supporting this argument is a single-centre retrospective study that compared 274 patients treated with uVATS to 448 treated with mVATS [13]. Propensity matching was performed and demonstrated fewer pneumonias, decreased intraoperative bleeding, shorter duration of surgery and hospital stay. A meta-analysis comparing mVATS and uVATS came to similar conclusions in that uVATS was associated with a reduction in overall complications, duration of chest-tube drainage and length of stay [14]. It should be noted that all studies included in the meta-analysis were observational and no randomized studies were available.

With respect specifically to pain and quality of life, there have been several studies suggesting superiority of uVATS to mVATS. Louis *et al.* published a single-centre study where all operations were performed by one surgeon. Patients with a history of opioid use were excluded, and the same postoperative analgesia protocol was used for all patients [15]. uVATS was associated with lower narcotic use in the recovery room, on postoperative days 1 and 2, and during the entire inpatient stay. Investigators from the Sichuan Cancer Hospital performed a comparative analysis from a longitudinal prospective study [16]. uVATS was associated with less pain during inpatient stay. The Violet study was a randomized trial comparing VATS to thoracotomy [9]. In the VATS group, a multiport approach was used in 166 participants and a uniportal approach in 42 participants. The uVATS approach was associated with better pain scores and physical function in a secondary analysis published from this trial [17].

Our study demonstrated an OS of 88% and a DFS of 76% at 3 years (Fig. 3), which compares favourably with the findings of Kang *et al.* in their study on uVATs lung resections. Kang *et al.* reported a 3-year OS of 82% (88% OS for stage I, 58.2% for stage I) and a 3-year DFS of 78% (86.2% for stage I, 59.5% for stage II) [18]. The study by Nachira *et al.*, who reported a 3-year OS of 78% for uVATS lobectomy compared to 74% for open surgery (*P* = 0.204), and a 3-year DFS of 62% in the uVATs group versus 66% in the open lobectomy group (*P* = 0.917) [19]. The study by Nachira *et al.* reinforces the oncological effectiveness of uVATs compared to open surgery, with no significant difference in postoperative complications, nodal upstaging, or mortality. Collectively, these studies highlight the promising mid-term oncologic and survival outcomes of the uniportal approach.

Our results indicate that uVATS lung resection is a safe and feasible technique. Adverse events, length of stay and chest tube duration were comparable to those reported in the literature for traditional mVATS and robotic VATS [20, 21]. There remain concerns about the technical challenges of uVATS with limited ability to articulate instruments and reduced spatial orientation compared to an mVATS. An experienced mVATS surgeon may be reluctant to see an increase in complication rates during the learning curve that will be required. Learning curve has been looked at by other investigators. Bedetti *et al.* analysed outcomes in 78 patients undergoing uVATS and compared their initial 30 patients to patients operated later in their series [22]. Operative time, conversion rate and length of stay was better in the latter group. In another series, a cut-off of 60 patients was used to define early experience [23]. Procedure time, blood loss, conversion rate to thoracotomy were all better after the initial learning experience in their study. The Uniportal VATS Interest Group of the European Society of Thoracic Surgeons published a consensus report on uVATS [24]. With respect to learning curve, 50 cases were felt to be the number required to overcome this. In our study, we saw no difference in outcomes over time with respect to complications, conversion to mVATS or open procedures, operative time and chest tube duration despite similar baseline characteristics between the groups. We did see a significant increase in the number of lymph node stations dissected, a decrease in the number of patients discharged with a chest tube, as well as a trend for more segmentectomies later in the series. Interestingly, there was an increase in length of stay from 3.3 days to 4.3 days. The reasons for the longer length of stay may have been related to a larger number of later procedures being performed at a community hospital where digital drainage systems were not available, and a smaller thoracic team with no advanced surgical fellows on the service making decisions to remove chest tubes. These results support the argument that with experience with mVATS, transition to uVATS can be safely undertaken. A similar finding was reported in another series by French *et al.* [25]. In their series, outcomes were compared between their first 50 uVATS procedures to 50 mVATS operations. There was no difference in conversions, complications and length of stay suggesting that safety, efficiency and quality were preserved when transitioning to a uVATS approach.

In North America, there has been increasing adoption and popularity of robotic lung resection [26]. It is possible that this will become the predominant approach for lung resection among young thoracic surgeons as they enter practice. Theoretically, pain would be expected to be similar for mVATS and multiportal robotic surgery, and a uniportal approach may be favourable. One study compared uVATS to robotic resection and demonstrated better pain control with uVATS at 1 week after operation, and similar pain scores at 2 and 3 weeks [27]. Physical function based on quality-of-life scores was also better after uVATS. The future may therefore be with uniportal robotic lung resection. Some early experience with this has been reported, and it will be interesting to see how this develops [28].

## CONCLUSION

While our study provides valuable insights into the early adoption of uVATS, it is important to acknowledge its limitations. The retrospective nature of the study, relatively small sample size and the single-centre setting may limit the generalizability of the findings. Additionally, the follow-up period could be extended to better assess long-term outcomes. Our study does however demonstrate the safety and feasibility of the uVATS approach as a valid option for thoracic surgeons. Additionally, the absence of a learning curve in our study supports consideration of this approach by thoracic surgeons who are experienced with multiportal VATS.

## Data Availability

Data cannot be shared for ethical/privacy reasons; we are restricted by institution.

## Notes

Presented at European Society of Thoracic Surgeons Annual Scientific Meeting, Barcelona, Spain, May 26–28, 2024.

## ABBREVIATIONS

DFS: Disease-free survival
mVATS: Multiportal video-assisted thoracic surgery
NSCLC: Non-small cell lung cancer
OS: Overall survival
uVATS: Uniportal video-assisted thoracic surgery
VATS: Video-assisted thoracic surgery
